# Setting up a surveillance system for sexually transmitted diseases in the general population with prospective data collection from private-practice and public-practice doctors in Hong Kong

**DOI:** 10.1186/1471-2458-11-254

**Published:** 2011-04-21

**Authors:** Joseph TF Lau, Chunqing Lin, King Man Ho, Man Chun Lau, Hi Yi Tsui, Jing Gu, Kuen Kong Lo

**Affiliations:** 1 Centre for Health Behaviours Research, School of Public Health and Primary Care, The Chinese University of Hong Kong, Hong Kong SAR, China; 2 Centre for Medical Anthropology and Behavioral Health, Sun Yat-sen University, Guangzhou, China; 3 Social Hygiene Service Headquarter, the Public Health Service Branch, Center for Health Protection, Department of Health, Hong Kong SAR, China; 4 Department of Medical Statistics and Epidemiology, School of Public Health, Sun Yat-sen University, Guangzhou, China

## Abstract

**Background:**

Existing surveillance systems for sexually transmitted diseases (STD) and reproductive tract infections (RTI) are important but often ineffective, as they tend to omit cases diagnosed by private-practice doctors

**Methods:**

During a 15-day study period, 277 private-practice doctors and all public-practice doctors of all the eight local Social Hygiene Clinics (SHC) in Hong Kong filled out daily a standard log-form, recording the number of patients diagnosed with particular types of STD/RTI. Projections for all local private-practice and public-practice doctors were made by the stratification method.

**Results:**

Data showed that 0.75% of private patients and 40.92% of public patients presented the listed STD/RTI syndromes. It is projected that 12,504 adults were diagnosed with such syndromes by all local private-practice (10,204) or public-practice doctors (2,300); 0.22% (male: 0.26%; female: 0.18%) of the local adult population would fall into this category. The ratio of STD/RTI cases, diagnosed by private-practice versus public-practice doctors, was 4:1. Of the participating private-practice doctors, 96% found the process easy to administer and 75% believed that it was feasible for such a STD/RTI surveillance system to be implemented annually.

**Conclusions:**

Surveillance of STD/RTI based only on data obtained from the public health system is inadequate. Data obtained from public-practice and private-practice doctors are very different and the majority of the patients presented their STD/RTI syndromes to private-practice doctors. The proposed, improved surveillance system is feasible and has the strengths of involving both private-practice and public-practice medical practitioners and being well accepted by private-practice doctors.

## Background

Accurate surveillance data on sexually transmitted diseases (STD) is crucial for the prevention, treatment and control of such diseases [1]. However, most of the current STD surveillance studies focus only on specific high risk groups [2], since population-based surveys are expensive and difficult to implement [3]. In many countries, case reporting remains the mainstay of surveillance on STD [4] and does not include data obtained from patients of private patients. In the United Kingdom, Italy and Portugal, only those STD cases seen by public-practice physicians need to be notified [4,5]. In mainland China, surveillance of STD relies solely on mandatory case-reporting data obtained from government facilities [5]. There is a need to improve the coverage - and hence the effectiveness - of the STD surveillance system [4]. Similarly in Hong Kong, surveillance data on STD are based on data reported by the Social Hygiene Clinics (SHC) of the Department of Health and patients who seek STD-related consultation from private-practice physicians are excluded from the surveillance system. In 2008, 13,867 STD cases were diagnosed in the SHC [6]. The ratio of STD cases being identified by local public-practice versus private-practice doctors remains unknown.

This study tested the feasibility of setting up a surveillance system for STD in the Hong Kong general population by projecting the number of STD cases presented to private-practice and public-practice doctors within a specific 15-day study period, in order to estimate territory-wide STD figures. The prevalence of STD and reproductive tract infection (RTI) syndromes among patients seeking consultations from three types of private-practice doctors, i.e., general practitioners (GP), obstetric and gynaecology (O&G) doctors and dermatologists/venerologists (D&V), and doctors of the public SHC, was estimated. Previous surveys revealed that these three types of doctors provided treatment for over 90% of the STD/RTI cases in Hong Kong [7,8]. The total numbers of patients presenting STD/RTI syndromes respectively to private-practice and public-practice doctors were projected. The ratio of such patients seen by the two types of doctors was calculated. In addition, the prevalence of all Hong Kong adults presenting STD/RTI syndromes to the two types of doctors during the study period was estimated.

## Methods

### Study population and sampling frames

The study population comprised all patients who sought medical consultations from private-practice GPs, O&G doctors, D&V doctors and doctors of the public SHC in Hong Kong from June 4 to June 18, 2007. The sampling frame of the D&V doctors was compiled using the membership list of the Hong Kong Association of Specialists in Dermatology; the sampling frames of the GP and O&G doctors were compiled from multiple sources, including websites of the Medical Council of Hong Kong and the Yellow Pages http://www.mchk.org.hk/doctor/index.htm and http://www.yp.com.hk/home/en/html/main/home.aspx and the Hong Kong Doctors Homepage http://www.hkdoctors.org. Contact information for 53 D&V doctors, 176 O&G doctors and 2644 GPs was thereby obtained. All listed GPs were grouped into 18 strata according to his/her clinic's district-board location. There were hence a total of 20 strata (18 GP strata + 1 O&G doctor stratum + 1 D&V doctor stratum). The Project was commissioned by the Department of Health in Hong Kong. Ethics approval was obtained from the Chinese University of Hong Kong. An enquiry hot-line was launched during the study period.

### Recruitment of doctors

All private-practice D&V doctors, O&G doctors and GPs listed in the constructed sampling frames were invited by mail and/or email to join the study. A follow-up telephone call was made one week afterwards. At least four other independent calls were made at different hours and on different days before an unanswered phone number was considered as invalid. A total of 895 GPs, 125 O&G doctors and 41 D&V doctors were contacted. Amongst these doctors with valid contact information, 247 GPs, 14 O&G doctors and 16 D&V doctors agreed to participate in this study: the response rates (defined as the number of doctors returning the log-form divided by the number of doctors with valid contact information) concerning the three types of private-practice doctors were respectively 27.6%, 11.2% and 39.0%. In addition, all public-practice doctors working in the eight SHCs participated in the study. Private-practice doctors who completed the study received a supermarket coupon (valued HK$200 or about 36US$) as a token of appreciation; Continuing Medical Education Units (CMEU) were also granted to participating private-practice doctors.

### Data collection

Daily during the 15-day data collection period, all participating doctors filled out a standard, anonymous log-form, recording the number of patients seen and the number of patients presenting STD/RTI syndromes under several categories, including urethral and vaginal discharge, genital ulcer, genital growth, ectoparastic infestations and other syndromes. Simple demographic information was collected. A research assistant telephoned the participating doctors to remind them to fill out and return the form and cross-checked the quality of the returned forms. Of all participating doctors, 96% returned all the required information during the study period.

### Data analysis

The total numbers of patients presenting specific STD/RTI syndromes to private-practice doctors, territory-wide during the 15-day period, was projected according to the stratification sampling method [9,10]. The projected number of patients presenting STD/RTI syndromes in a particular stratum was calculated by the following formula: N _projected number of patients in the stratum _= (Total number of doctors in the stratum/Number of participating doctors in the stratum) * N _observed number of patients in the stratum_

It was assumed that the participating clinics within a stratum was a representative sample of all the clinics in their respective stratum and that the three types of private-practice doctors were seeing almost all the STI/RTI cases in Hong Kong. The number of patients presenting the specific types of syndromes during the study period, territory-wide in the private-practice sector was then estimated by totaling the figures of all the 20 strata. Bootstrap methods [11] (30,000 bootstrap replications) were used to estimate the 95% confidence intervals for the total number of patients presenting particular syndromes in all strata and in the Hong Kong territory by using MATLAB V7.1 (the MathWorks, Inc.).

An estimate of the prevalence of STD/RTI among patients seeking medical consultation from private-practice and public-practice doctors was calculated by the actual number of patients with STI/RTI, divided by the total number of patients seeking medical consultations from the sampled doctors during the study period. The prevalence of adults in the Hong Kong general population who presented STD/RTI syndromes to private-practice and public-practice doctors during the study period was estimated as the projected territory-wide number of adults with STD/RTI syndromes (diagnosed by private-practice and public-practice doctors during the study period), divided by the total number of people aged 18 and above (2006 By-census: 5,657,031; 2,650,340 males and 3,006,691 females).

## Results

### Doctors' and patients' characteristics

Of the 277 participating doctors, 230 (83%) were male. The geographic location (D&V: p = 0.33, O&G: p = 0.38 and GP: p = 0.11, chi-square test) and gender (D&V: p = 0.92, O&G: p = 0.08, chi-square test) of the participating doctors were not statistically different from those of the doctors who declined to participate in this study. Most of the patients seen by the O&G doctors were female (96%) and most of the patients seen by the D&V and SHC doctors were male (90% and 81.6%), whilst the sex ratio of those seen by GP was close to 1.0 (Table 1). Respectively, 33.4% and 20.9% of all the private patients and SHC patients belonged to the 31-40 age-group.

**Table 1:** Demographic characteristics of patients with STD/RTI syndromes who sought care in private and public sectors

	No. of patients seen by General Practitioners (N = 660)		No. of patients seen by Obstetrics & Gynecology specialists (N = 81)		No. of patients seen by Dermatology & Venereology specialists (N = 100)		No. of patients seen by all private-practice doctors (N = 841)		**No. of patients seen by public-practice SHC doctors**† (N = 2,311)		χ^2^ (private vs. public)
	n	%	n	%	n	%	n	%	n	%	p-value
Gender											
Male	326	49.4	3	3.7	90	90.0	419	49.8	1886	81.6	<0.001
Female	326	49.4	78	96.3	10	10.0	414	49.2	422	18.3	
Missing	8	1.2	0	0.0	0	0.0	8	1.0	3	0.1	
Age group											
<18	4	0.6	0	0.0	0	0.0	4	0.5	11	0.5	<0.001
18-30	174	26.4	30	37.0	14	14.0	218	25.9	492	21.3	
31-40	223	33.8	28	34.6	30	30.0	281	33.4	482	20.9	
41-50	161	24.4	17	21.0	28	28.0	206	24.5	514	22.2	
>50	82	12.4	3	3.7	28	28.0	113	13.4	666	28.3	
Missing	16	2.4	3	3.7	0	0.0	19	2.3	146	6.3	

**†**Data on 1 particular day were missing in 2 male SHC doctors: estimates for that day were made by entering the average of the 14-days data of the 2 particular SHC.

### Prevalence of patients presenting STD/RTI syndromes whilst seeking medical consultation from the specific types of doctors

The prevalence of STD/RTI syndromes (of any type) among all private patients was 0.75% (GP: 0.63%, O&G: 3.94%, D&V: 1.83%; Table 2), as compared to the 40.92% for the SHC patients. Urethral/vaginal discharge was the modal category of STD/RTI syndromes presented by private patients (prevalence = 0.45%), whilst genital growth was the modal category presented by SHC patients (prevalence = 25.61%). The distributions of syndromes presented by the D&V patients were similar to those presented by the SHC patients, whilst they were different from those presented by the GP patients.

**Table 2:** Pattern of STD/RTI syndromes among patients who sought care in private and public sectors

	No. of patients seen by General Practitioners (N = 104,933)		No. of patients seen by Obstetrics & Gynecology specialists (N = 2,057)		No. of patients seen by Dermatology & Venereology specialists (N = 5,475)		No. of patients seen by all private-practice doctors (N = 112,465)		**No. of patients seen by public-practice SHC doctors**† (N = 5,647)	
	n	%*	n	%*	n	%*	n	%*	n	%*
% presenting STD/RTI syndromes#										
Urethral/vaginal discharge	428	0.41	66	3.21	8	0.15	502	0.45	437	7.74
Genital ulcer	71	0.07	2	0.10	25	0.46	98	0.09	218	3.86
Genital growth	74	0.07	7	0.34	52	0.95	133	0.12	1,446	25.61
Ectoparasitic infestations	10	0.01	0	0.00	1	0.02	11	0.01	29	0.51
Other syndromes	126	0.12	13	0.63	17	0.31	156	0.14	262	4.64
										
Had any one of the above syndromes	660	0.63	81	3.94	100	1.83	841	0.75	2,311	40.92

* The percentages of the particular STD/UTI syndromes were calculated by the number of patients with the particular syndromes, divided by the total number of patients who sought medical care during the study period. If the total number of patients who sought medical care on a particular day was not provided by the doctor (121 missing entries for GP, 20 for O&G, 18 for Dermatologist and 2 for SHC), estimate on this figure was obtained by taking the average of all doctors in the particular group.

# Patients may have more than 1 symptom.

† Data on 1 particular day were missing in 2 male SHCs: estimates for that day were made by entering the average of the 15-days data of the 2 particular SHC.

### Projected number of patients presenting STD/RTI syndromes to private-practice and public-practice doctors in Hong Kong during the study period

The projected territory-wide numbers of private patients presenting different types of syndromes were: 6,261 (urethral and vaginal discharge: 1720 males and 4484 females), 1,154 (genital ulcer: 977 males and 177 females), 1,305 (genital growth: 996 males and 303 females), 151 (ectoparastic infestations: 100 males and 51 females), 2,028 (other syndromes: 1511 males and 466 females) and 10,204 (any of the above-mentioned types: 5003 males and 5093 females, with 95% CI: 9,420 to 10,994; Table 3). Corresponding figures obtained from the SHC were respectively 434, 216, 1,446, 29, 260 and 2,300. The private-to-public ratios for these studied types of patients were hence 14:1 (urethral/vaginal discharge), 5:1 (genital ulcer), 1:1 (genital growth), 5:1 (ectoparasitic infestation) and 8:1 (other syndromes). The private-to-public ratio for 'any of the aforementioned categories' was around 4.4:1 (10,204:2,300).

**Table 3:** Projected number of patients presenting studied STD syndromes to private-practice and public-practice doctors during the study period by gender aged 18 or above

	Private-practice doctors				Public-practice doctors			All				Private to public ratio		
	
	Male	Female	Total	95% CI	Male	Female	Total	Male	Female	Total	95% CI	Male	Female	Total
% presenting STD/RTI syndromes #														
Urethral/vaginal discharge	1720	4484	6261	(5363-6984)	285	149	434	2005	4633	6695	(6073-7314)	6:1	30:1	14:1
Genital ulcer	977	177	1154	(884-1442)	192	24	216	1169	201	1370	(1088-1646)	5:1	7:1	5:1
Genital growth	996	303	1305	(1028-1602)	1249	191	1446	2245	494	2751	(2391-2965)	1:0.8	2:1	1:1
Ectoparasitic infestations	100	50	151	(56-267)	22	7	29	122	57	180	(84-295)	5:1	7:1	5:1
Other syndromes	1511	466	2028	(1672-2395)	190	68	260	1701	534	2288	(1917-2640)	8:1	7:1	8:1
														
Had any one of the above syndromes	5003	5093	10204	(9420-10994)	1881	416	2300	6884	5509	12504	(11598-13172)	3:1	12:1	4:1

Remarks: Data on 1 particular day were missing in 2 male SHCs: estimates for that day were made by entering the average of the 15-days data of the 2 particular SHC.

### Estimated prevalence of Hong Kong adults presenting STD/RTI syndromes to private-practice and public-practice doctors during the 15-day study period

The results are summarized in Table 4. The total population size of the adult population (age ≥18 years old) was used as the denominator of these estimations. The prevalence of the adult general population diagnosed to present specific types of syndromes to private-practice and public-practice doctors over the study period was: 0.0012 (urethral and vaginal discharge: 0.0008 for males and 0.0015 for females), 0.0002 (genital ulcer: 0.0004 for males and 0.00007 for females), 0.0005 (genital growth: 0.0008 for males and 0.0002 for females), 0.00003 (ectoparastic infestations: 0.00005 for males and 0.00002 for females), 0.0004 (other syndromes: 0.0006 for males and 0.0002 for females) and 0.0022 (any of the above-mentioned types: 0.0026 for males and 0.0018 for females). Again, these figures are not prevalence of STD/RTI in the general population and they are to be used for surveillance purpose.

**Table 4:** Prevalence of Hong Kong adults (> = 18 years old) presenting STD/RTI to public-practice or private-practice doctors during June 4-18

	Male	Female	Total
Urethral/vaginal discharge	0.0008	0.0015	0.0012
Genital ulcer	0.0004	0.00007	0.0002
Genital growth	0.0008	0.0002	0.0005
Ectoparasitic infestations	0.00005	0.00002	0.00003
Other syndromes	0.0006	0.0002	0.0004
			
Had any one of the above syndromes	0.0026	0.0018	0.0022

Remarks: Data on 1 particular day were missing in 2 male SHCs: estimates for that day were made by entering the average of the 15-days data of the 2 particular SHC

### Process evaluation

A self-administered process-evaluation questionnaire survey was completed by the 247 participating doctors, with a response rate of 89% (247/277). The majority of the participating private-practice doctors (96%) found the log-form easy to complete and indicated that they would participate in similar studies in the future, whilst over 75% believed that it would be feasible to implement similar studies to collect STD data from private-practice doctors on an annual basis.

## Discussion and conclusions

The study demonstrated the feasibility of setting up an improved STD/RTI surveillance system based on the number of cases being diagnosed by private-practice and public-practice doctors in Hong Kong over a defined time period. The process evaluation results are encouraging. It is hence possible to create a stable cohort of participating doctors, allowing the Department of Health to trace the prevalence of STD/RTI in the general population. The results obtained from this study can be used as baseline data for future surveillance studies using the same methodology.

In countries such as the United Kingdom, Italy, China and Portugal, surveillance of STD are still dependent on reporting of cases via government channels [4,5]. Our study demonstrated that such surveillance systems might provide incomplete or even inaccurate STD data, as private and public patients have rather different socio-demographic characteristics and presented different types of syndromes during the clinical visit. Only two out of nine patients presenting STD/RTI syndromes sought consultation from public-practice doctors: however, the ratio varied from 1:1 for genital growth to 14:1 for urethral/vaginal discharge (i.e. those with urethral/vaginal discharge were quite unlikely to see public-practice doctors). Case-reporting from government medical services alone is inadequate to form an adequate STD surveillance system.

It is projected that, within the 15-day study period, about 12,504 adult STD/RTI cases were presented to all private-practice or public-practice doctors in Hong Kong. The estimated prevalence of adult STD/RTI diagnosed by all private-practice and public-practice doctors during the study period was 0.22%. It must be understood that this figure is not the prevalence of STD/RTI among adults in Hong Kong; asymptomatic STD/RTI cases such as Chlamydia were not included in the numerator. The figure could at best be seen as a lower limit of the prevalence of STD/RTI. The purpose of this study was, instead, to establish a viable surveillance system that can capture changes over time.

In this study, the sex ratio of patients presenting STI/RTI syndromes in public SHC was much lower than that of the private patients; 55.7% and 72.1% of the patients with urethral/vaginal discharge were females in the public and private clinics respectively. The observed contrast might be explained by differing health-seeking behaviors by gender. Women are significantly less likely to seek treatment from public hospitals or genital-urinary medical clinics [12-14]. The higher level of stigmatization associated with women contracting STD might compromise their access to information and power of decision on seeking health care, as compared to their male counterparts [14,15]. Females might also perceive stronger stigmatisation when presenting STI/RTI syndromes to public-practice doctors; they may therefore resort to private-practice doctors, who may be able to offer a better sense of privacy and confidentiality and hence a low degree of stigmatisation. Potential obstacles to females in utilizing public STD services should be reviewed and be rectified, as women who could not afford private services may have their STI/RTI syndromes untreated, or may utilize over-the-counter medicine to treat such problems [12].

The fact that the majority of the STI/RTI cases presented to public-practice doctors were male may lead to a misconception that STI/RTI in Hong Kong is essentially a problem of the male, which is not true. It is observed that, although males were more likely than females to present genital growth, ectoparasitic infestations and other syndromes to private-practice and public-practice doctors, the reverse was true for syndromes including urethral/vaginal discharge and genital ulcer. Overall, males have a higher prevalence of STD/RTI than females, but the difference was only moderate (0.26% versus 0.18%). Taking private-practice and public-practice doctors' cases into account, a very different conclusion about sex differentials would be made, as compared to that based only on private-practice doctors' data.

The response rates of the O&G and D&V doctors were low. Provision of a higher monetary incentive and collaborations with the professional associations might improve the response rates. According to the projected number, about 4% of the O&G doctors' patients presented some STD/RTI syndromes to their doctors. These doctors should therefore be involved regularly in safer sex promotion campaigns. This is especially relevant to female patients, who are much less likely than males to make use of public STD services. It is seen that 70.8% of all STD patients that were captured from this study came from the GP. It is therefore important for public-practice health workers to develop partnerships with private-practice doctors and to strengthen health promotion for HIV/STD prevention in the primary care environment.

The study has several limitations: First, despite multiple means of providing incentives to participate in the study, the overall response rate for the three types of private-practice doctors was only 26%. We therefore have to assume that there was no difference between the numbers of STD/RTI patients seen by doctors who did and did not participate in the study. Although the geographical locations and genders of the participating doctors were comparable to those doctors who declined to participate, this assumption could still be invalid. Other information, such as the sizes of clinics, was not available for comparison. The direction of the potential bias is unknown. Second, only three categories of private-practice doctors were included in this study. Other specialists may also have provided care to STD/RTI cases during the study period, although it is expected that such numbers would be relatively small. Third, the study period was short and the choice of timing was arbitrary; the results may be biased by seasonal effects. Fourth, several studies demonstrated that a large proportion of patients used self-medication rather than licensed health facilities to treat their STD syndromes and these cases were not covered by this study [12,16,17]. Increase in this form of health service-seeking behavior among STD patients may therefore confound observed trends. Additionally, asymptomatic cases were also not included in this study; the size of such population is unknown. However, the proposed study design may be acceptable from a surveillance perspective, as the method would still be able to detect differences and trends, even though the level may have been shifted by some biases, assuming the biases and the pattern of health-seeking behaviors among STI/RTI patients remain rather stable over time. Lastly, data were self-reported by the doctors and issues surrounding accuracy of information may exist. Validation of such data was not feasible.

In summary, despite the aforementioned limitations, our study offers a viable and pioneering approach for setting up a surveillance system by including data from both private and public practitioners. The private-practice doctors found this system acceptable to them. The data have important policy implications for STD prevention and control in Hong Kong. It is necessary that private-practice doctors participate in STD/RTI surveillance and contribute to HIV/STD prevention. The public STD services should also cater for the gender-specific needs of patients.

## Competing interests

The authors declare that they have no competing interests.

## Authors' contributions

JL is the Principal Investigator of the project and the lead writer of the manuscript. CL helped to draft the manuscript and assisted in the statistical analysis. KMH and MCL were involved in the design and implementation of the study and editing of the manuscript. HYT, KKL and JG proposed suggestions to improve the study and revised the manuscript. All authors read and approved the final manuscript.

## Pre-publication history

The pre-publication history for this paper can be accessed here:

http://www.biomedcentral.com/1471-2458/11/254/prepub
